# Characterization and Antimicrobial Resistance Profiles of Biofilm Forming Strains of *Staphylococcus aureus* Isolated from Skin Lesions

**DOI:** 10.3390/microorganisms13112449

**Published:** 2025-10-25

**Authors:** Nikola Dančová, Ján Király, Vanda Hajdučková, Patrícia Hudecová, Simona Hisirová, Mária Nagyová, Zuzana Fedáková, Emil Pilipčinec, Gabriela Gregová

**Affiliations:** 1 Department of Public Veterinary Medicine and Animal Welfare, The University of Veterinary Medicine and Pharmacy in Košice, 041 81 Košice, Slovakia; nikola.dancova@uvlf.sk; 2 Department of Microbiology and Immunology, The University of Veterinary Medicine and Pharmacy in Košice, 041 81 Košice, Slovakia; jan.kiraly@uvlf.sk (J.K.); vanda.hajduckova@uvlf.sk (V.H.); patricia.hudecova@student.uvlf.sk (P.H.); simona.hisirova@student.uvlf.sk (S.H.); emil.pilipcinec@uvlf.sk (E.P.); 3 Department of Medical and Clinical Microbiology, Faculty of Medicine, Pavol Jozef Šafárik University in Košice, Trieda SNP 1, 040 11 Košice, Slovakia; maria.nagyova@upjs.sk; 4 Department of Dermatovenerology, Faculty of Medicine, Pavol Jozef Šafárik University in Košice, Trieda SNP 1, 040 11 Košice, Slovakia; zuzana.fedakova@upjs.sk

**Keywords:** *Staphylococcus aureus*, skin lesions, biofilm formation, antimicrobial resistance, efflux pump

## Abstract

*Staphylococcus aureus* is an important human pathogen known for its versatility and ability to cause a wide range of infections. The aim of this study was to isolate and identify *S. aureus* from skin lesions from human patients, to determine antimicrobial resistance and biofilm formation potential at phenotypic and genotypic levels, as well as to verify the activity of efflux pump production. Out of 51 samples collected from skin lesions of various etiologies, 13 isolates were identified as *S. aureus*. All isolates showed the ability to form biofilms, which correlated with the presence of the *icaABCD*, *agrA*, *srtA*, *clfAB*, and *fnbAB* genes, while the *bap* gene was absent. The highest rates of resistance were observed for ampicillin (69.2%) and gentamicin (46.2%), as well as for erythromycin and clindamycin (38.5%). The *mecA* gene was present in two isolates, but phenotypic resistance to methicillin was confirmed in only one of them, suggesting possible heterogeneous expression or regulated activity of resistance mechanisms. The *mecC* gene was not present in any isolate. Efflux pump production was observed in only three isolates, showing weak to intermediate levels. These findings indicate the high biofilm potential and variable antimicrobial resistance of *S. aureus* clinical isolates, which pose a challenge for the treatment of emerging skin infections.

## 1. Introduction

The skin is the largest organ of the human body and performs several key functions, such as protective, thermoregulatory, and sensory functions. Skin diseases encompass a variety of disorders that impact the skin and its related structures and are among the most common diseases in the human population worldwide [1,2]. Among these, a skin lesion—defined as an abnormality or change in the structure, appearance, or colour of the skin that differs from the surrounding tissue—can be superficial or extend into deeper parts. We distinguish between primary (present from birth or arising during life) and secondary (changes in the primary lesion), and between benign and malignant skin lesions. Their emergence is often the result of the interaction of various internal and external factors [1,3].

The most commonly occurring skin lesions are skin and soft tissue infections caused by a wide range of microorganisms [2]. Under normal conditions, the skin hosts a diverse microbiome that is in balance. However, when the skin’s natural defence mechanisms are weakened [4] or the integrity of the skin barrier is compromised, it creates conditions for the colonisation of the affected area by a variety of microorganisms [5]. The most commonly isolated pathogens of skin and soft tissue infections include *Staphylococcus aureus*, *Streptococcus* spp., *Clostridium* spp., *Enterococcus* spp., *Klebsiella pneumoniae*, *Escherichia coli*, *Proteus* spp., *Pseudomonas aeruginosa*, *Acinetobacter baumannii*, *Neisseria* spp., and *Vibrio* spp. [6,7].

*Staphylococcus aureus* (*S. aureus*) is commonly found in the physiological microbiota of humans and animals but is also a highly adaptable and opportunistic pathogen capable of causing various diseases. Its pathogenicity is driven by a complex of virulence factors that enhance its capacity to adhere to surfaces, invade or evade the host’s immune system, or generate substances that harm tissues [8]. Proteins referred to as MSCRAMM (microbial surface components recognizing adhesive matrix molecules), which include the clustering factors ClfA (*clfA*), ClfB (*clfB*), fibronectin-binding proteins FnbA (*fnbA*), FnbB (*fnbB*), or proteins composed of serine-aspartate dipeptide repeats (SdrC, SdrD, SdrF), contribute not only to adhesion but also to invasion of host cells or tissues [9]. Concomitantly secreted toxins such as α-hemolysin, Panton-Valentine leukocidin (PVL), or toxic shock syndrome toxin (TSST) contribute to tissue destruction and systemic toxicity [10]. Overexpression of the *srtA* gene, which encodes the transpeptidase sortase A, enhances the speed at which surface proteins are anchored during cell wall biosynthesis, leading to heightened virulence [9].

*S. aureus* can form biofilms on medical devices, prosthetic implants, and tissue surfaces, protecting the bacteria from the immune system and antimicrobial treatments and contributing to chronic or recurrent infections [11]. These biofilms are typically encased in a glycocalyx layer composed of polysaccharide intercellular antigen (PIA), synthesised by the *icaABCD* locus, though some strains form biofilms independently of this locus. Other factors involved in biofilm formation include protein A, fibronectin-binding proteins, autolysin, extracellular DNA, and biofilm matrix proteins such as Bap [10]. The *agrA* gene encodes a regulatory factor that is part of the *agr* (accessory gene regulator) quorum-sensing system, a key regulatory mechanism in *S. aureus* that controls the expression of virulence factors, biofilm formation, and also influences the ability of the bacterium to cause disease [12].

The treatment of infections caused by *S. aureus* remains challenging due to the rapid development of resistance to antimicrobial agents. The occurrence of methicillin-resistant *S. aureus* (MRSA) in both healthcare and community environments worsens these issues through its various resistance mechanisms, including the production of penicillin-binding protein 2a (PBP2a), which is encoded by the *mecA* gene found within the staphylococcal chromosome cassette *mec* (SCC*mec*). The global rise of resistant strains, including MRSA, vancomycin-intermediate *S. aureus* (VISA), and vancomycin-resistant *S. aureus* (VRSA), underscores the pathogen’s remarkable evolutionary adaptability [13].

Another important mechanism of antimicrobial resistance in *S. aureus* is the activation of efflux pumps [14]. These pumps are transport proteins found in the cytoplasmic membrane of bacteria and play a critical role in excreting drugs from the cell [15]. They also have a regulatory function—they are involved in the secretion of toxic substances into the external environment [16]. According to the energy source, they are divided into primary, where the source is ATP, and secondary, where the source is an electrochemical gradient [17]. There are six families of efflux pumps; each family is characterised by a group of substrates. In *Staphylococcus* species, four of these families—ABC (the ATP-binding cassette family), MATE (the multidrug and toxin extrusion family), MFS (the major facilitator superfamily family), and SMR (the small multidrug resistance family)—have been documented. Notably, the first three families are associated with antimicrobial resistance [18,19].

This study aimed to identify and monitor the prevalence of *S. aureus* from skin lesions of different etiologies obtained from hospitalized patients. The study included monitoring biofilm formation as a significant virulence factor associated with antimicrobial resistance, detecting antimicrobial resistance profiles, assessing the production of efflux pumps, and identifying genes related to antimicrobial resistance as well as genes involved in biofilm formation. The main objective of this study was to obtain and provide new insights into the phenotypic and genotypic profiles of antimicrobial resistance and biofilm formation.

## 2. Materials and Methods

### 2.1. Sample Collection, Cultivation, and Identification of Staphylococcus aureus

Over a six-month period, a total of 51 samples from skin lesions of various etiologies were obtained from patients hospitalised at the Department of Dermatovenerology of the University of Pavol Jozef Šafárik and the Louis Pasteur University Hospital in Košice, Slovakia. The samples were taken from lesions with a confirmed infectious etiology, based on clinical evaluation (presence of pus, blisters, rashes, spots, wounds, bleeding, swelling, erythema, local warmth, pain, or itching). One swab was collected from each patient using a sterile cotton swab with Amies Copan 108C transport medium (Copan Italia, Brescia, Italy). Samples were stored at 4 °C before culture on media and processed within 24 h of collection.

All samples were grown on the surface of Columbia Blood Agar with the addition of 5% sterile defibrinated sheep blood, Baird-Parker Agar, and Mannitol Salt Agar (HiMedia Laboratories, Mumbai, India) and incubated under the conditions listed in Table 1. The isolated bacteria were categorised into the *Staphylococcus* genus based on their growth traits, biochemical characteristics, and Gram staining.

Species identification was performed using the commercial biochemical kit STAPHYtest 24 (Erba Lachema, Brno, Czech Republic). All identified staphylococci were confirmed at the genus level through multiplex PCR (mPCR) with primers that amplify a segment of the 16S rRNA gene specific to the *Staphylococcus* genus. Additionally, species-specific primers for *S. aureus* were used to identify the genes *eap* (extracellular adhesion protein) and *nuc* (thermostable nuclease). Finally, all staphylococcal isolates that were biochemically identified and confirmed at the genetic level using mPCR were also identified using a proteomic approach with MALDI-TOF MS (version 2.0, BioTyper Library version 3.0; Bruker Daltonics, Billerica, MA, USA) [20].

### 2.2. Genomic DNA Extraction

Genomic DNA was extracted from overnight cultures of *S. aureus* inoculated in modified brain heart infusion broth (mBHI; HiMedia Laboratories, Mumbai, India) containing 1.0% glucose and 2.0% sodium chloride using the High Pure PCR DNA Extraction Kit (Roche Molecular Systems, Inc., Pleasanton, CA, USA). The quantity and purity of DNA were analyzed using a NanoDropTM spectrophotometer with an ND-8000 system (Thermo Fisher Scientific, Waltham, MA, USA) by measuring absorbance at 260 and 280 nm.

### 2.3. Testing of Biofilm Formation

The ability of *S. aureus* isolates to form biofilms was evaluated using a modified method based on O’Toole et al. [21]. Each well of a 96-well polystyrene microtiter plate F-type (Brand GMBH + CO KG, Wertheim, Germany) received 100 μL of mBHI and 100 μL of a bacterial suspension at 1 McFarland turbidity. The plates were incubated stationary at 37 °C for 24 h. After incubation, the contents of the wells were aspirated, and 200 μL of a 0.1% crystal violet solution was added to each well, followed by 30-min incubation at room temperature. After washing and drying the wells, crystal violet that had bound to the adherent cells was extracted with 200 μL of 30% acetic acid per well. *S. epidermidis* CCM 4418, a non-biofilm-forming strain, and *S. aureus* CCM 4223, a biofilm-forming strain (Czech Collection of Microorganisms, Brno, Czech Republic), were used as reference strains. The negative control was pure mBHI. The optical density was determined spectrophotometrically at a wavelength 550 nm using a BioTek model Synergy 4 reader (Merck, Darmstadt, Germany) in triplicate for each strain tested, and then the mean values were calculated.

### 2.4. Antimicrobial Susceptibility Testing

The minimum inhibitory concentration (MIC) testing was determined by the colourimetric microdilution method according to Gattringer et al. [22] with automated readout via the Miditech system (Bel-Miditech s.r.o., Bratislava, Slovakia). The following antimicrobials were tested: ampicillin (AMP), ampicillin + sulbactam (SAM), piperacillin + tazobactam (TZP), oxacillin (OXA), cefoxitin (FOX), gentamicin (GEN), ciprofloxacin (CIP), moxifloxacin (MFX), erythromycin (ERY), clindamycin (CLI), linezolid (LNZ), rifampicin (RIF), vancomycin (VAN), teicoplanin (TEC), tetracycline (TET), tigecycline (TGC), chloramphenicol (CHL), trimethoprim (TMP), trimethoprim + sulphonamide (COT), and nitrofurantoin (NIT). The results of the MIC values of each antimicrobial were interpreted according to the clinical breakpoints of EUCAST 2024 (version 14.0) [23].

### 2.5. Detection of Efflux Pump

Efflux pump production activity was assessed on Mueller-Hinton agar (HiMedia Laboratories, Mumbai, India) containing 2.5 mg/L ethidium bromide in a Petri dish using a cartwheel method based on expelling a fluorescent dye, as described by Martins et al. [24]. The plates were divided into eight sectors by radial lines (cartwheel pattern). A bacterial suspension (0.5 McFarland standard) was prepared from the overnight culture and saline solution. The samples were swabbed onto agar surfaces, beginning at the middle and progressing toward the periphery. Subsequently, the plates were incubated for 24 h at 37 °C, protected from light in aluminium foil. After incubation, the swabbed plates were examined by the UV-Reader Quantum system (source of the UV light; Vilber Lourmat, Collégien, France) and analysed with the VisionCapt digital imaging system (Vilber Lourmat, Collégien, France). *S. aureus* ATCC 25923EtBr (efflux pump producer) and *S. aureus* ATCC 25923 (non-efflux pump producer) from the American Type Culture Collection (Manassas, VA, USA) served as reference strains. Saline solution alone was used as a negative (purity) control.

### 2.6. Gene Detection Using PCR

Genes associated with antimicrobial resistance, specifically *mecA* and *mecC* (which encode resistance to beta-lactams), as well as genes involved in biofilm formation (including *bap*, *icaABCD*, *clfAB*, *fnbAB*, *srtA*, and *agrA*), were detected using both simplex and mPCR. Table 2 provides a list of all primers used in the PCR reaction. The PCR reactions were carried out in a Mastercycler^®^ Nexus X2 thermal cycler (Eppendorf, Hamburg, Germany). The composition of the reaction mixture and the PCR conditions used in this study were described by Király et al. [25]. Amplified PCR products were electrophoretically separated in a Wide Mini-Sub^®^ GT Cell electrophoresis system (Bio-Rad, Hercules, CA, USA) on a 2.5% agarose gel using the non-toxic GoodView™ reagent (Amplia s.r.o., Bratislava, Slovakia). Subsequently, PCR products were visualised under UV light of an ultraviolet transilluminator (Bio-Imaging Systems, Modi’in-Maccabim-Re’ut, Israel) and recorded using a Kodak Gel Logic 100 digital imaging system (Kodak, Rochester, NY, USA).

### 2.7. Statistical Analysis

Biofilm formation was evaluated based on the obtained data by statistical analysis with GraphPad Prism program version 8.3.0 (GraphPad Software Inc., San Diego, CA, USA) through One-Way ANOVA, accompanied by Dunnett’s test to assess significance at *p* < 0.001.

The minimum inhibitory concentration values were further processed using the Miditech Analyser interpretation software (Bel-Miditech s.r.o., Bratislava, Slovakia, cat. n. 002002), which also performs statistical analysis.

## 3. Results

### 3.1. Identification of Bacterial Isolates

Out of 51 examined samples collected from skin lesions of hospitalised patients, 29 samples were identified as representatives of the genus *Staphylococcus* based on culture, biochemical characteristics, and microscopic examination.

Using the STAPHYtest 24 biochemical kit, 29 isolates of *Staphylococcus* spp. were identified, with 13 classified as *S. aureus* and 16 as coagulase-negative staphylococci (CoNS).

All staphylococci were subsequently confirmed using mPCR. The 29 isolates were positive for the presence of a section of the 16S rRNA gene specific for the genus *Staphylococcus* (141 bp). Additionally, fragments of the *eap* (230 bp) and *nuc* (103 bp) genes were detected in the identified *S. aureus* strains (13/29), which were absent in the other *Staphylococcus* spp. (16/29). This confirmed the results obtained at the phenotypic level.

All staphylococci isolates were also identified using MALDI-TOF MS. According to the obtained score values, the staphylococci were identified as *S. aureus* (*n* = 13), while 16 CoNS isolates included the following species: *S. epidermidis* (8/16), *S. haemolyticus* (7/16), and *S. capitis* (1/16).

### 3.2. Evaluation of Biofilm Formation

**Figure 1:**
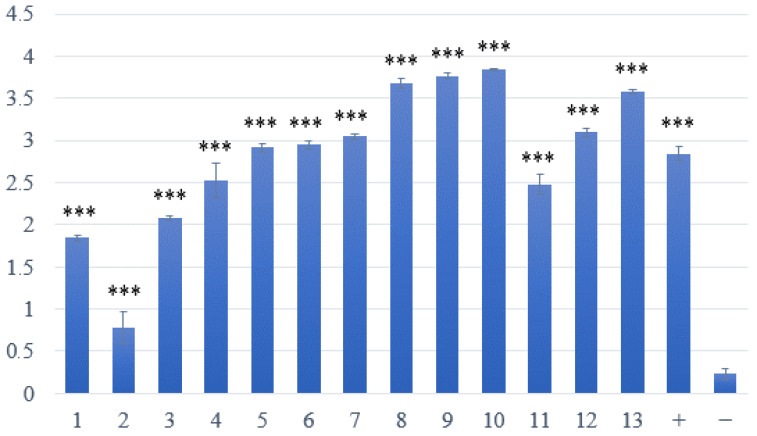
Assessment of biofilm formation in *S. aureus* isolated from skin lesions. Samples 1–13—strains with weak or strong production of biofilm; positive (+) control—biofilm-forming *S. aureus* CCM 4223; negative (−) control—non-biofilm-forming *S. epidermidis* CCM 4418. *** Significantly higher ability to produce biofilm (*p* < 0.001).

The ability to form biofilms was evaluated in all 13 confirmed isolates of *S. aureus*. All isolates demonstrated significant biofilm-forming ability, alongside a positive control (*S. aureus* reference strain CCM 4223), in comparison to the non-biofilm-forming reference strain *S. epidermidis* CCM 4418. One isolate (sample no. 2) exhibited a notably lower ability for biofilm formation than the other isolates (Figure 1).

### 3.3. Antimicrobial Susceptibility Profiles of S. aureus Isolates

The minimum inhibitory concentration (MIC) of antimicrobials was determined in 13 confirmed isolates of *S. aureus* (Table 3).

*S. aureus* isolates were predominantly susceptible to all antimicrobial agents tested. Among the 13 total isolates, the highest resistance rates were observed to ampicillin (69.2%), gentamicin (46.2%), erythromycin, and clindamycin (38.5%). Resistance to oxacillin, cefoxitin, and chloramphenicol was recorded in one isolate (7.7%).

The MIC xG values for erythromycin were 1.30 mg/L, for clindamycin 0.80 mg/L, and for erythromycin 8.9 mg/L, which exceeded the EUCAST clinical breakpoints.

**Figure 2:**
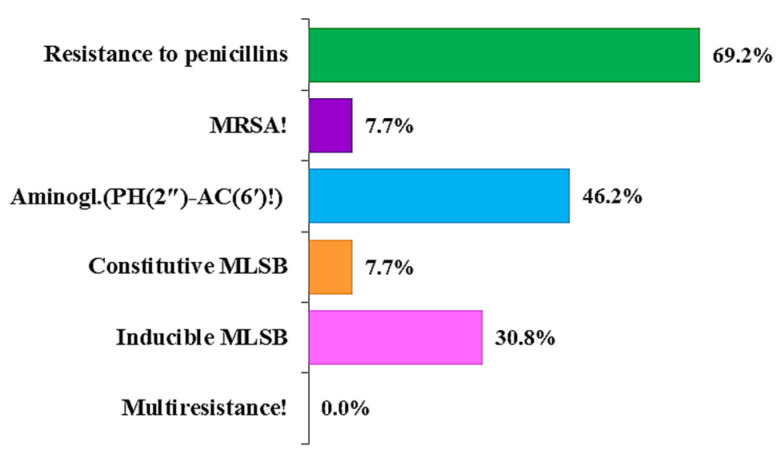
Overview of phenotypically predicted resistance mechanisms in clinical *S. aureus* isolates.

According to the resistance profile, the system automatically evaluated the resistance mechanisms (Figure 2). It is important to note that multiple resistance mechanisms have been identified in some strains simultaneously. The most common mechanism was resistance to penicillins (69.2%), followed by resistance to aminoglycosides (PH(2″)-AC(6′)!) (46.2%); this represents complications in treatment with these antimicrobial agents and combined enzymatic resistance to gentamicin, tobramycin, netilmicin, and ampicillin. The MLSB (macrolide-lincosamide-streptogramin B) mechanism was also present. The MLSB inducible (MLSB/i) (30.8%), manifested in clinical resistance to lincosamides and streptogramin B induced by 14- and 15-membered macrolides, constitutive (MLSB/c) (7.7%), determining resistance to all MLSB antimicrobials, were found [32]. One isolate (no. 11; 7.7%) was confirmed to have an MRSA mechanism, in which all beta-lactams are clinically ineffective in therapy. Multiresistance was not detected in any of the *S. aureus* isolates studied.

Although the system did not evaluate multiresistance in any of the isolates examined, based on the analysis of the susceptibility profile to the tested antimicrobial substances, we found that isolates 4, 5, 7, 12, and 13 exhibited multiresistance (Table 3), which we define as the resistance of bacteria to three or more different groups of antimicrobial substances.

### 3.4. Evaluation of Efflux Pump Production

**Figure 3:**
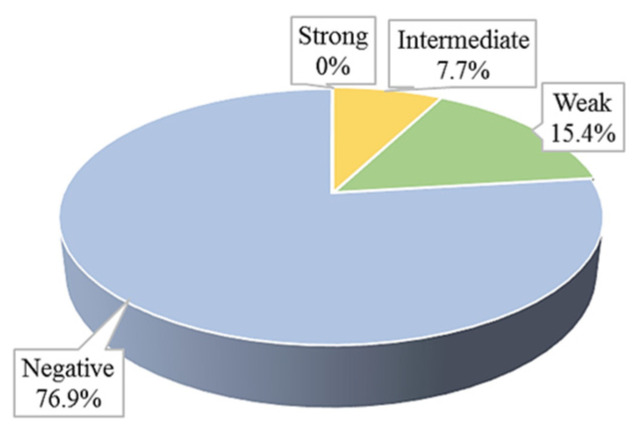
Efflux pumps production in clinical *S. aureus* isolates.

**Figure 4:**
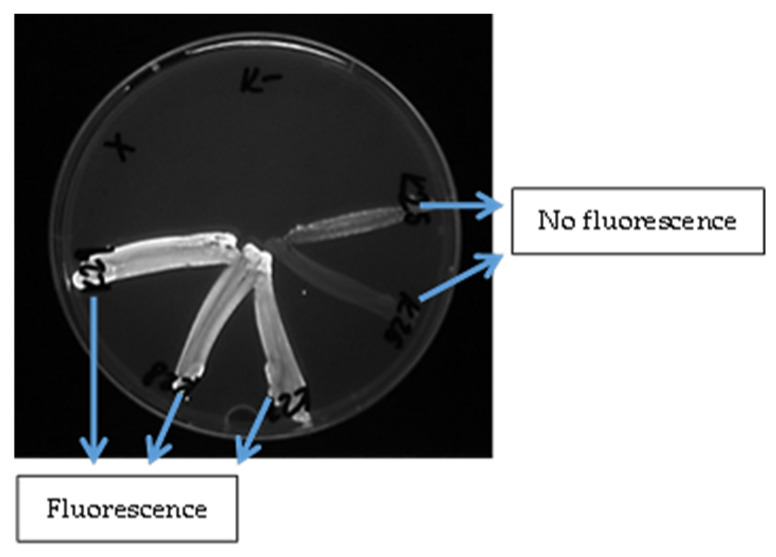
Evaluation of the fluorescence of *S. aureus* isolates detected under UV light after adding ethidium bromide to the medium.

Out of the 13 isolates tested, none were classified as strong producers of efflux pumps. One isolate (7.7%) was categorised as an intermediate efflux pump producer. Two isolates (15.4%) were identified as weak efflux pump producers, while the remaining ten isolates (76.9%) showed no signs of efflux pump production (Figure 3 and Figure 4).

### 3.5. Identification of Antimicrobial Resistance Genes and Genes Related to Biofilm Formation

In our study, we analysed 13 *S. aureus* isolates and detected the *mecA* gene in only two clinical samples (isolates no. 4 and 11). Of these, only sample number 11 was identified as MRSA, exhibiting phenotypic resistance to the beta-lactam antibiotics ampicillin, oxacillin, and cefoxitin. Additionally, the *mecC* gene was not found in any of the isolates we examined, which aligns with its lower prevalence in clinical practice.

A more detailed analysis of biofilm formation was performed in all 13 isolates, not only at the phenotypic level but also at the genotypic level, using mPCR and simplex PCR (gene *bap*). All *S. aureus* isolates showed the ability to form biofilms, which correlated with the high presence of *icaABCD*, *agrA*, *srtA*, and also *fnbAB* and *clfAB* genes encoding adhesive proteins. The *bap* gene was absent in all of the isolates; nevertheless, this does not eliminate the possibility of considerable biofilm activity, since biofilm formation in *S. aureus* is affected by a range of intricate mechanisms. A summary of the biofilm-related genes detected in *S. aureus* can be found in Table 4. Table 5 presents a detailed evaluation of clinical strains of *S. aureus*.

## 4. Discussion

Due to its characteristic properties, human skin has unique properties that make it an unsuitable environment for many microorganisms, effectively acting as a barrier that prevents their entry into the organism. Healthy skin is naturally dry, has a low pH on the surface, and an upper layer that continuously renews itself. The skin also produces antimicrobial peptides that can directly destroy or inhibit the growth of microorganisms. However, certain bacteria, such as *Staphylococcus* spp., particularly *S. aureus*, can overcome these defence mechanisms and not only colonise the skin but also be the primary cause of complicating pathogenesis and persistence of skin diseases [33].

In our study, we identified *S. aureus* in 25.5% (13/51) of clinical samples collected from skin lesions of various etiologies in hospitalised patients. All isolates were confirmed using both molecular and proteomic methods, ensuring high accuracy in identification at the species level. Our findings are consistent with previously published data indicating that *S. aureus* is a significant etiological agent responsible for skin and soft tissue infections, especially in patients with weakened immunity or compromised skin barrier [5,6]. The rate of *S. aureus* colonisation of skin lesions in published studies varies not only between countries but also between cities where the studies were conducted. In the study by Khalili et al. [34], the colonisation rate of *S. aureus* skin lesions was 45%, with results varying between cities (Shiraz 23% vs. Tehran 51%). A higher prevalence is also reported by Vella et al. [35], who also recorded a higher presence in the cities of Chicago (92%), Columbia (44%), and Vanderbilt (40%). The authors state that differences in results could be related to sampling methods, repeated infections, and variations among healthcare facilities. On the other hand, similar or lower prevalence rates were observed in studies from Nigeria (26.6%) [36], Ethiopia (23.6%) [37], and Brazil (20%) [38].

Biofilm formation is a significant virulence factor that complicates the treatment of skin infections caused by *S. aureus*. The ability of bacteria to form resilient biofilms may lead to their persistent presence on the human skin. Additionally, *S. aureus* bacteria within biofilms are shielded from the host’s immune response and the effects of antimicrobial agents, which can result in recurrent and chronic infections [39,40]. In a study conducted in Sweden, a total of 160 clinical isolates of *S. aureus* from patients with various skin diseases were examined, and all of them successfully formed biofilms [41]. Our results showed that all 13 *S. aureus* isolates analysed also had the ability to form biofilm, which correlates with the presence of key biofilm-related genes.

The *icaABCD* operon was found in all isolates, underscoring its central function in the production of polysaccharide intercellular adhesin (PIA), a crucial component of the biofilm matrix. Among the genes involved, *icaA* and *icaD* are particularly important for the biofilm formation process [11]. Moreover, all isolates were also positive for *agrA* and *srtA*, which are genes involved in regulating virulence factor expression and attachment of surface proteins. These findings further support the fact that biofilm formation in *S. aureus* is multifactorial and carefully regulated by quorum sensing and protein anchoring systems [9]. In our study, we detected the adhesive genes *clfA*, *clfB*, *fnbA*, and *fnbB* at varying frequencies. These genes play a crucial role in the initial adhesion to host tissues and the accumulation of biofilms [8]. Although the bap gene was absent in all isolates, it is important to note that the presence of the *bap* gene is not universal across all strains, and this absence does not preclude the possibility of biofilm formation. Biofilm formation can occur independently of Bap, using alternative mechanisms that involve other adhesins such as *icaABCD* or *fnbAB*. This suggests that there may be functional redundancy or species-specific pathways in biofilm formation [11,42].

The *bap* gene encodes the Bap (biofilm-associated protein) protein, a high-molecular-weight protein that was first described in *S. aureus* isolated from bovine mastitis [43]. Bap is crucial for biofilm formation, particularly in facilitating initial attachment to both living (biotic) and non-living (abiotic) surfaces. The C-terminal region of Bap contains a typical domain involved in cell adhesion. Moreover, it enhances strong intercellular adhesion when the Bap protein experiences partial proteolytic cleavage, which releases fragments that contain the N-terminal region. In this reaction, which is influenced by environmental factors like calcium ion concentration and acidic pH, spontaneous aggregation into amyloid fibers occurs. The gene is commonly located on mobile genetic elements, which leads to its varying distribution among different isolates. All *S. aureus* isolates that express Bap exhibit high adhesion and strong biofilm-forming ability [11,44,45].

The prevalence of detected biofilm-associated genes (such as *bap*, *icaABCD*, *clfAB*, *fnbAB*, *srtA*, and *agrA*) differs substantially across published studies, reflecting variability in the distribution and detection rates of individual genes [29,46,47,48].

Resistant bacteria, particularly multiresistant strains, pose a major public health problem. Among these, MRSA is one of the most significant regarding resistance to antimicrobial agents [6]. Recent studies indicate a decrease in the prevalence of MRSA in hospitals; however, it remains a significant cause of skin and soft tissue infections. Community-associated MRSA (CA-MRSA) appears to be more virulent and spreads more rapidly than hospital-associated MRSA (HA-MRSA) [49]. Higher levels of resistance among groups of people with poor hygiene, limited access to healthcare, and those engaging in risky behaviours (i.e., injecting drugs) contribute to the persistence and spread of MRSA in the population. Therefore, it is important to focus not only on appropriate therapeutic approaches but also on prevention and education in the areas of hygiene and antimicrobial resistance [50].

In our study, the most dominant resistance of *S. aureus* isolates was to ampicillin (69.2%) and also to oxacillin (7.7%), which belong to the beta-lactam group. This finding is consistent with other published studies, which suggest a near-universal prevalence due to the widespread production of beta-lactamases encoded mainly by the *blaZ* gene [51]. Koumaki et al. [52] indicate that the prevalence of *S. aureus* resistance to beta-lactams continues to be significantly high in both community and hospital settings, frequently surpassing 90%. Similarly, in a study by Ji et al. [51], *S. aureus* from patients with skin and soft tissue infections also showed dominant resistance to penicillin and oxacillin (100%), followed by cefoxitin (95.2%). They also found resistance to clindamycin and erythromycin in *S. aureus*, but only in 76.2% of the isolates examined. In contrast, resistance to gentamicin was less than 14%. No isolate was resistant to daptomycin, ceftaroline, linezolid, tigecycline, teicoplanin, or vancomycin.

Our results also showed increased resistance to gentamicin (46.2%) and erythromycin (38.5%), highlighting the need for more cautious use of these antimicrobial agents. The prevalence of clindamycin resistance among *S. aureus* isolates was 38.5% in our study. A recent systematic review reported that the prevalence of clindamycin resistance in *S. aureus* isolates varies significantly, ranging from 2.9% to 44%. The highest prevalence, at 44%, was reported in Egypt, while the lowest prevalence of 2.9% was observed in Côte d’Ivoire. These findings highlight the need for a more rational and locally adapted use of this antimicrobial [53,54]. Previous research has shown an increased occurrence of multidrug-resistant *S. aureus* in both community and hospital settings [6].

According to our results, the most frequently detected resistance mechanisms in *S. aureus* isolates were resistance to penicillins and to aminoglycosides caused by production of modification enzyme PH(2″)-AC(6′)!. Resistance to penicillins is generally caused by the production of beta-lactamases or the presence of the *mecA* gene, which encodes the penicillin-binding protein PBP2a. PBP2a prevents beta-lactam from binding to the cell surface due to reduced affinity [55]. In 2011, a new *mecC* gene was discovered, which shows 63% homology with the *mecA* gene. The *mecC* gene encodes the PBP2c protein, which gives staphylococci a similar level of resistance to beta-lactams as PBP2a does. These mechanisms lead to resistance against virtually all beta-lactams, a phenomenon known as MRSA [56]. Our study confirmed the presence of one isolate of MRSA at the phenotypic level, and it was found to carry the *mecA* gene. This finding aligns with the results of Almuhayawi et al. [7], who reported increased rates of methicillin and vancomycin resistance among *S. aureus* isolates from skin lesions. On the other hand, the *mecC* gene was not present in any of the isolates, confirming its lower incidence in clinical settings [13]. In addition to PBP2a/PBP2c synthesis, staphylococcal resistance to beta-lactams may be caused by other mechanisms. Some MRSA strains are associated with so-called resistance mechanisms without *mec* genes. Examples include strains with BORSA (borderline oxacillin-resistant *S. aureus*) or MODSA (modified *S. aureus*) phenotypes [56].

The Miditech system has also identified the mechanism of MLSB resistance—acquired resistance to macrolides, lincosamides, and streptogramin B. This type of resistance results from reduced binding of these drugs to the methylated 50S ribosomal subunit, which is an overlapping binding site for all three groups of antimicrobial agents [57]. There are two types of MLSB resistance: constitutive MLSB phenotype (MLSB/c), in which the bacterial strain constantly produces rRNA methyltransferase, and inducible MLSB (MLSB/i), in which methyltransferase production is observed in the presence of an inducing agent [58]. Establishing the MLSB phenotype accurately is crucial, given that clindamycin is advised for managing uncomplicated skin and soft tissue infections, particularly as an alternative for treating MRSA-related infections [59]. The research conducted by Henry and Khachemoune [60] confirmed the MLSB phenotype in seven strains, of which three exhibited resistance to methicillin at the same time. Current resistance to beta-lactams, macrolides, and lincosamides greatly restricts available treatment options. Likewise, this study identified three isolates that exhibited simultaneous resistance to aminoglycosides along with MLSB resistance, further complicating the management of these infections. However, resistance to rifampicin, tetracycline, and ciprofloxacin was not observed, which are frequently employed for treating skin infections in human medicine [61].

Most articles describing efflux pumps are devoted to reviews, or rather genotypic characterisation, and the impact of new potential efflux pump inhibitors (e.g., [62,63,64]). From the available data about phenotypic identification of efflux pumps, Favour et al. [65] in their study report up to 83% occurrence of efflux pump producers in their samples. However, they were only multiresistant isolates without distinction into weak, intermediate, and strong producers. Nevertheless, it is more compared to our 23.1%. Patel et al. [66] identified efflux pumps in 47.7% of isolates, which is also more than we managed to do. The reason may also be the larger number of clinical isolates of *S. aureus*. Abdi et al. [67] described a 10% incidence of clinical isolates of *S. aureus* producing efflux pumps.

## 5. Conclusions

The findings of our study substantiate the clinical relevance of *Staphylococcus aureus* as a cause of skin infections of various etiologies, particularly due to its strong ability to form biofilms and increasing antimicrobial resistance. All isolates displayed phenotypic capacity for biofilm formation, which is closely related to the presence of key genes such as *icaABCD*, *agrA*, *srtA*, *clfAB*, and *fnbAB*. While the bap gene was absent, biofilm formation was nonetheless strong, underscoring the intricate nature of regulatory mechanisms. The observed antimicrobial resistance, including multiresistant and MRSA strains, highlights the need for targeted diagnostics and treatment. The results support the importance of combining phenotypic and genotypic methods in assessing the virulence and resistance of clinical isolates of *S. aureus*. 

## Figures and Tables

**Table 1:** Microbiological media, incubation conditions, and criteria for identification *S. aureus*.

Microbiological Medium	Incubation Conditions			Colony Appearance
	Temperature	Time	Atmosphere	
Columbia Blood Agar	35–37 °C	24–48 h	Aerobic	Golden-yellow, white, or cream colonies with beta/gamma hemolysis
Baird-Parker Agar	30–35 °C	24–48 h	Aerobic	Grey-black and shiny colonies with a positive, opaque zone around the colony
Mannitol Salt Agar	30–35 °C	18–72 h	Aerobic	Yellow or white colonies surrounded by a yellow zone

**Table 2:** List of primers used for PCR analysis.

Gene	Primer Sequence (5′→3′)	Product Size (bp)	Reference
*16S rRNA*	TACAATGGACAATACAAAGGGC TCACCGTAGCATGCTGATCT	141	[26]
*eap*	TACTAACGAAGCATCTGCC TTAAATCGATATCACTAATACCTC	230	[27]
*nuc*	ACCTGCGACATTAATTAAAGCG TGTTTCAGGTGTATCAACCAATAATAG	103	[25]
*mecA*	TGGAAGTTAGATTGGGATCATAGC CGATGCCTATCTCATATGCTGTT	154	[25]
*mecC*	GACGATGGATCTGGTACAGCA CATTCATGAATGGATAAACATCGTA	94	[25]
*bap*	TTGACGAGGTTGGTAATGGC CGCCTACAGTTTCTGGTAATGC	87	[25]
*icaA*	CTTGCTGGCGCAGTCAATAC GTAGCCAACGTCGACAACTG	75	[28]
*icaB*	ATACCGGCGACTGGGTTTAT ATGCAAATCGTGGGTATGTGT	141	[29]
*icaC*	CTTGGGTATTTGCACGCATT GCAATATCATGCCGACACCT	209	[30]
*icaD*	ACCCAACGCTAAAATCATCG GCGAAAATGCCCATAGTTTC	211	[29]
*srtA*	GTGGTACTTATCCTAGTGGCAGC GCCTGCCACTTTCGATTTATC	183	[31]
*agrA*	TCGTAAGCATGACCCAGTTG AAATCCATCGCTGCAACTTT	96	[31]
*fnbA*	GAAGTGGCACAGCCAAGAAC ACGTTGACCAGCATGTGG	192	[31]
*fnbB*	CAATGATCCTATCATTGAGAAGAGTG CCTTCTACACCTTCAACAGCTGTA	156	[31]
*clfA*	GAGAGCATTTAGTTTAGCGGCA TCACCTTTAACAGCAGAATTAGGC	180	[25]
*clfB*	GTCTACACAAACGAGCAATACCAC TGAGGAACAGTTTGATCTTGCA	120	[25]

**Table 3:** Antimicrobial susceptibility profile of *S. aureus* isolates.

No. of Isolates													
	1	2	3	4	5	6	7	8	9	10	11	12	13
AMP	0.25	0.25	8	0.5	4	0.5	0.5	0.25	0.25	1	16	4	4
SAM	0.25	1	4	0.25	2	0.25	0.25	0.25	0.25	0.25	4	2	2
TZP	0.5	0.5	1	1	2	0.5	0.5	0.5	0.5	4	8	2	4
OXA	0.25	0.5	0.5	0.25	0.25	0.5	0.25	0.25	0.25	0.5	8	0.5	0.5
FOX	2	4	4	4	8	4	4	4	4	2	32	4	4
GEN	0.5	0.5	0.5	0.5	128	0.5	16	0.5	128	128	0.5	128	16
CIP	0.25	0.5	0.5	0.5	0.25	0.25	0.5	1	0.5	0.5	0.125	0.5	2
MFX	0.063	0.125	0.125	0.125	0.063	0.063	0.125	0.063	0.031	0.063	0.031	0.25	0.125
ERY	0.5	0.25	0.5	8	16	0.25	0.25	16	0.25	0.25	0.25	16	16
CLI	0.25	0.25	0.25	1	8	0.125	0.125	1	0.125	0.25	0.1255	1	1
LNZ	4	2	2	2	4	2	2	2	2	4	2	2	2
RIF	0.063	0.031	0.031	0.031	0.031	0.031	0.031	0.031	0.031	0.063	0.031	0.031	0.031
VAN	1	0.5	1	1	1	1	1	1	2	1	0.5	2	2
TEC	1	1	0.5	0.5	0.5	0.5	0.5	0.25	0.25	0.5	0.25	2	0.5
TET	0.25	0.25	0.25	0.125	0.125	0.25	1	1	0.125	0.25	0.125	0.25	0.25
TGC	0.063	0.063	0.063	0.063	0.063	0.063	0.125	0.063	0.063	0.125	0.063	0.125	0.063
CHL	4	4	4	4	4	4	64	8	4	8	4	4	4
TMP	1	4	2	1	1	2	2	0.5	1	1	1	2	1
COT	0.25	0.25	0.25	0.125	0.25	0.5	0.25	0.25	0.25	0.25	0.125	0.25	0.25
NIT	8	16	8	8	8	8	8	8	8	8	8	8	16

Description: AMP = ampicillin, SAM = ampicillin + sulbactam, TZP = piperacillin + tazobactam, OXA = oxacillin, FOX = cefoxitin, GEN = gentamicin, CIP = ciprofloxacin, MFX = moxifloxacin, ERY = erythromycin, CLI = clindamycin, LNZ = linezolid, RIF = rifampicin, VAN = vancomycin, TEC = teicoplanin, TET = tetracycline, TGC = tigecycline, CHL = chloramphenicol, TMP = trimethoprim, COT = trimethoprim + sulphonamide, NIT = nitrofurantoin. Values highlighted in grey represent resistance to the given antimicrobial agents.

**Table 4:** Presence and distribution of genetic determinants of biofilm formation in *S. aureus* isolates.

Gene	No. of Positive Isolates (*n* = 13)	% of Occurrence
*icaA*, *icaB*, *icaC*, *icaD*	13/13	100.0
*agrA*	13/13	100.0
*srtA*	13/13	100.0
*fnbA*	6/13	46.2
*fnbB*	3/13	23.1
*clfA*	9/13	69.2
*clfB*	13/13	100.0
*bap*	0/13	0.0

Description: *n* = number of isolates. Explanations of gene abbreviations are provided in the section “Materials and Methods”.

**Table 5:** Comprehensive evaluation of clinical *S. aureus* strains: phenotypic antimicrobial resistance and biofilm formation, efflux pump data, and genotypic analysis.

No. of Isolates	Antimicrobial Resistance	Resistance Mechanism	Biofilm Formation	Efflux Pump Production	Occurrence of Genes
1	-	-	Strong	Negative	*icaABCD*, *agrA*, *srtaA*, *fnbB*, *clfAB*
2	-	Resistance to penicillins	Weak	Negative	*icaABCD*, *agrA*, *srtA*, *fnbA*, *clfAB*
3	AMP	Resistance to penicillins	Strong	Negative	*icaABCD*, *agrA*, *srtA*, *clfAB*
4	AMP, ERY, CLI	Resistance to penicillins; Inducible MLSB/i	Strong	Weak	*mecA*, *icaABCD*, *agrA*, *srtA*, *clfB*
5	AMP, GEN, ERY, CLI	Resistance to penicillins; Aminogl.PH(2″)-AC(6′)!; Constitutive MLSB/c	Strong	Intermediate	*icaABCD*, *agrA*, *srtA*, *fnbAB*, *clfAB*
6	AMP	Resistance to penicillins	Strong	Negative	*icaABCD*, *agrA*, *srtA*, *fnbB*, *clfAB*
7	AMP, GEN, CHL	Resistance to penicillins; Aminogl. PH(2″)-AC(6′)!	Strong	Weak	*icaABCD*, *agrA*, *srtA*, *fnbA*, *clfAB*
8	ERY, CLI	Inducible MLSB/i	Strong	Negative	*icaABCD*, *agrA*, *srtA*, *clfB*
9	GEN	Aminogl.PH(2″)-AC(6′)!	Strong	Negative	*icaABCD*, *agrA*, *srtA*, *fnbA*, *clfAB*
10	AMP, GEN	Resistance to penicillins; Aminogl.PH(2″)-AC(6′)!	Strong	Negative	*icaABCD*, *agrA*, *srtA*, *fnbA*, *clfAB*
11	AMP, OXA, FOX	MRSA!	Strong	Negative	*mecA*, *icaABCD*, *agrA*, *srtA*, *fnbA*, *clfAB*
12	AMP, GEN, ERY, CLI	Resistance to penicillins; Aminogl.PH(2″)-AC(6′)!; Inducible MLSB/i	Strong	Negative	*icaABCD*, *agrA*, *srtA*, *clfB*
13	AMP, GEN, ERY, CLI	Resistance to penicillins; Aminogl.PH(2″)-AC(6′)!; Inducible MLSB/i	Strong	Negative	*icaABCD*, *agrA*, *srtA*, *clfB*

## Data Availability

The original contributions presented in this study are included in the article. Further inquiries can be directed to the corresponding author.

## Abbreviations

The following abbreviations are used in this manuscript:

*agr*	accessory gene regulator
AMP	ampicillin
BORSA	borderline oxacillin-resistant *Staphylococcus aureus*
CA-MRSA	community-associated methicillin-resistant *Staphylococcus aureus*
CIP	ciprofloxacin
CLI	clindamycin
CoNS	coagulase-negative staphylococci
COT	trimethoprim + sulphonamide
DNA	deoxyribonucleic acid
ERY	erythromycin
EUCAST	European Committee on Antimicrobial Susceptibility Testing
FOX	cefoxitin
GEN	gentamicin
HA-MRSA	hospital-associated methicillin-resistant *Staphylococcus aureus*
CHL	chloramphenicol
LNZ	linezolid
MALDI-TOF MS	matrix-assisted laser desorption ionization–time of flight mass spectrometry
mBHI	modified brain heart infusion broth
MFX	moxifloxacin
MIC	minimum inhibitory concentration
MLS_B_	macrolide-lincosamide-streptogramin B resistance
MODSA	modified *Staphylococcus aureus*
MRSA	methicillin-resistant *Staphylococcus aureus*
MSCRAMM	microbial surface components recognizing adhesive matrix molecules
NIT	nitrofurantoin
OXA	oxacillin
PCR	polymerase chain reaction
PIA	polysaccharide intercellular antigen
PVL	Panton-Valentine leucocidin
RIF	rifampicin
SAM	ampicillin + sulbactam
SCC*mec*	staphylococcal cassette chromosome *mec*
TEC	teicoplanin
TET	tetracycline
TGC	tigecycline
TMP	trimethoprim
TSST	toxic shock syndrome toxin
TZP	piperacillin + tazobactam
VAN	vancomycin
VISA	vancomycin-intermediate *Staphylococcus aureus*
VRSA	vancomycin-resistant *Staphylococcus aureus*
